# The efficacy of kinesiology taping for improving gross motor function in children with cerebral palsy: A systematic review

**DOI:** 10.4102/sajp.v74i1.459

**Published:** 2018-08-29

**Authors:** Marianne Unger, Juan P. Carstens, Natasha Fernandes, Rulanda Pretorius, Suzelle Pronk, Ashleigh C. Robinson, Kara Scheepers

**Affiliations:** 1 Division of Physiotherapy, Stellenbosch University, South Africa

## Abstract

**Background:**

Kinesiology taping is an increasingly popular technique used as an adjunct to physiotherapy intervention for children with cerebral palsy (CP), but as yet we do not have a review of the available evidence as to its efficacy.

**Objectives:**

To critically appraise and establish best available evidence for the efficacy of truncal application of kinesiology taping combined with physiotherapy, versus physiotherapy alone, on gross motor function (GMF) in children with CP.

**Method:**

Seven databases were searched using the terms CP, kinesio taping and/or kinesiology tape and/or taping, physiotherapy and/or physical therapy and GMF. Only randomised controlled trials (RCTs) were included and appraised using the PEDro scale. Revman© Review Manager was used to combine effects for GMF in sitting, standing and activities of daily living.

**Results:**

Five level IIB RCTs that scored 3–6/8 on the PEDro scale were included. Meta-analysis showed that taping was effective for improving GMF in sitting and standing as measured by the Gross Motor Function Measure (B) (*p* < 0.001) and (D) (*p* < 0.001), respectively.

**Conclusion:**

There is moderate evidence to support kinesiology taping applied to the trunk as an effective intervention when used as an adjunct to physiotherapy to improve GMF in children with CP, especially those with GMF Classification Scale levels I and II, and particularly for improving sitting control.

**Clinical implications:**

Kinesiology taping is a useful adjunct to physiotherapy intervention in higher functioning children with CP. Current evidence however is weak and further research into methods of truncal application is recommended.

## Introduction

Children with cerebral palsy (CP) typically present with motor impairments including tone abnormalities, muscle weakness and increased reflexes (Bax et al. 2005) that lead to postural balance deficits and coordination problems adversely affecting self-care, mobility, social functioning and participation (Rosenbaum et al. 2007). There are numerous interventions for the treatment of CP-associated impairments, but unfortunately, the evidence for many of these is scant. Novak et al. (2013) reported in their systematic review (SR) low to no conclusive evidence for most interventions used within standard care, including physiotherapy techniques such as manual stretching and neurodevelopmental therapy. This, and given the different types and wide range of clinical presentations of CP, makes therapeutic intervention decision-making for therapists difficult.

One intervention not included in Novak et al.’s (2013) review was that of kinesiology taping (KT) – an increasingly popular technique used in both child and adult rehabilitation (Morris et al. 2013). Kinesiology tape is a thin, elastic therapeutic tape applied directly onto the skin and consists of an air permeable, water-resistant cotton matrix that can stretch longitudinally with a stretch capacity of 40% – 60% of its resting length, mimicking human skin properties (Morris et al. 2013). It is hypothesised that KT application may enhance muscle and myofascial functions and influence cutaneous mechanoreceptors by providing constant afferent stimulation. This allows more sensory information to flow to the central nervous system for integration in the presence of mechanical loads, resulting in improved voluntary control and coordination (Morris et al. 2013). Kinesiology taping has been shown to stimulate muscle activity, support weak muscles and provide proprioceptive feedback in adults to maintain postural alignment in both the healthy population (Kahanov 2007), in persons with neuro-musculoskeletal pathology (Jaraczewska & Long 2006; Thelen et al. 2008) and in those with hemiplegia (Al-Shareef, Omar & Ibrahim 2016).

Considering that sensory and proprioceptive feedback are prerequisites for proper motor development (Hadders-Algra 2000), taping could be an effective intervention strategy in paediatric rehabilitation. The results of studies investigating the effect of KT in children have, however, reported mixed outcomes. A pilot trial in four children with hemiplegic CP found an improvement in dynamic activities, but not during static functional activities when Kinesio Taping^®^ was applied to the lower limb (Da Costa et al. 2013). Similar outcomes were reported in a study that found that functional taping in children with CP had no effect on postural sway measurements during quiet stance (Pavão et al. 2017). In another study, Kinesio Taping^®^ was applied to both the upper and lower limbs in children with CP and a significant improvement in physical fitness and gross motor function (GMF) was found (Kaya Kara et al. 2015). In a more recent study, the authors reported an increased activity in the rectus femoris muscle following taping applied to the lower limb in children with unilateral CP, but no improvement in functional ability (Dos Santos et al. 2018). In another study that applied taping across the paraspinal muscles in children with spastic quadriplegia, no effect was found for improved GMF (Footer 2006). An SR by Guchan and Mutlu (2016) who looked specifically at the effects of taping in children with CP concluded that the evidence for taping in this population remains inconclusive. Cunha et al.’s (2017) SR also concluded that the evidence remains scant for children with disabilities, including those with CP. Both reviews commented that there is some evidence, but the taping protocols varied so between studies – ranging from limb taping to truncal taping and using rigid to elastic taping – weakening the evidence for this potentially useful adjunct to the current standard of care.

Because of its proposed effects, relatively inexpensive cost and easy application, KT may be an effective complementary adjunct to current physiotherapy interventions for improving GMF in children with CP. However, the quality of the available evidence supporting KT in this context has not yet been well established. Contradictory findings warrant further investigation and a more thorough review. The purpose of this SR was thus to critically appraise and collate the best available evidence for the effectiveness of KT (as opposed to rigid taping) applied to the trunk as an adjunct to physiotherapy, versus physiotherapy alone, for improving GMF in children with CP. This SR could possibly enhance evidence-based practice in the field of CP, to enable physiotherapists to make more informed decisions regarding optimal treatment with KT in this population.

## Methodology

### Search strategy

Seven electronic databases, accessed through the Stellenbosch University library services, were searched from inception to May 2018 (Cochrane Library, EBSCO Host [CINAHL, Pre-CINAHL], Google Scholar, PEDro, PubMed, Science Direct and Scopus). Individual search strategies were developed for each database according to its function. Key search terms included CP, physiotherapy, physical therapy, kinesiology taping, KT taping, kinesio tape, taping and GMF. Each database was searched independently by two authors. Retrieved titles, abstracts and full texts were scrutinised independently by each member of the group of authors based on the eligibility criteria set at the onset of the review. Through discussion within the group, the final articles for full review were selected.

### Study inclusion and exclusion criteria

Studies were assessed according to the following eligibility criteria:

Type of studies: Only randomised controlled trials (RCTs) published in English and scoring three or more on the PEDro scale (De Morton 2009) were considered for inclusion.

Participants: Studies were included if they recruited male and/or female children (< 18 years old), diagnosed with CP but otherwise healthy. Studies were excluded if they involved participants who previously participated in trials using KT, had undergone any orthopaedic surgery or had received botulinum toxin injection in the 6 months prior to the assessment date, had structural scoliosis or demonstrated an allergic reaction to any materials used in the study.

Interventions: Kinesiology taping applied to the trunk as an adjunct to conventional physiotherapy (including, but not confined to, neurodevelopmental treatment [NDT], constraint-induced manual therapy [CIMT], stretching, muscle strengthening, tone modulation exercises, gait re-education and balance re-education exercises). Studies using rigid taping or any other forms of taping not conforming to the specific properties of KT were excluded.

Comparisons: Conventional physiotherapy without any KT application.

Outcomes: Studies were included if they used outcome measures assessing GMF – including, but not limited to, motion analysis, the Gross Motor Function Measure (GMFM), Paediatric Balance Scale (PBS), Timed-Up-And-Go (TUG), Bruininks–Oseretsky Test of Motor Proficiency (BOTMP) and Sitting Assessment Scale (SAS).

### Evidence hierarchy and methodological appraisal

The National Health and Medical Research Council (NHMRC) Hierarchy of Evidence (Merlin, Weston & Tooher 2009) was used to rank the level of evidence of each article. Methodological quality of each included article was appraised using the PEDro scale. The scale appraises internal validity and statistical reporting according to 11 criteria and is a valid and reliable assessment of the methodological quality of clinical trials (de Morton 2009). For the purpose of this review, it was considered acceptable to omit the two criteria related to blinding of the participant (criterion 5) and therapist (criterion 6) as, given the nature of the intervention (KT), this would be difficult to do. The criterion related to tester blinding, however, was included. Each article was assigned to two authors who independently appraised the article. Results were compared and a third author was consulted in the event of any discrepancies.

### Data extraction method

The adapted Joanna Briggs Institute data extraction form was used to extract and capture the relevant data from the included articles. Data were obtained concerning the following categories: citation, study type, participants, interventions, comparisons, outcome measures, results, post-intervention clinical status and implications. Two articles were allocated to each author to perform data extraction, ensuring that information could be cross-checked and unanimity reached among the authors with subsequent recompilation of the data.

### Data analysis

Data pertaining to GMF in sitting and in standing were synthesised in the form of meta-analyses using the Revman^©^ Review Manager Software (2008) using a fixed-effects approach to illustrate combined effects in the form of forest plots. Weighted mean differences (WMDs) were used to express outcomes for continuous data (mean and standard deviation [SD]) and the *I*^2^ statistic was used to assess heterogeneity among the studies. Where it was not possible to pool the data, a narrative description of findings is presented.

### Ethical considerations

Ethical approval for this study was not required as all data used were publicly available. However, all studies included in this review had ethical clearance.

## Results

### Search strategy

The results of the search strategy are summarised in Table 1. The initial search yielded a total of 1951 hits. Seven potentially eligible full-text articles were evaluated according to the eligibility criteria, whereafter five articles (Badawy, Ibrahem & Shawky 2015; Ibrahim 2015; Karabay et al. 2016; Kaya Kara et al. 2015; Şimşek et al. 2011) were accepted for inclusion in this review. Reasons for excluding articles included study designs other than RCTs and the use of taping other than KT.

**TABLE 1:** Article identification search strategy.

Databases or other sources	Initial hits	Accepted titles	Accepted abstracts
Cochrane	285	4	4
Ebsco Host-CINAHL	863	5	4
PEDro	7	1	2
PubMed	473	5	3
Science Direct	142	17	0
Google Scholar	125	19	4
Scopus	56	8	5
**Total**	**1951**	**54**	**22**

Note: Duplicates between the databases = 17

### Evidence hierarchy and methodological appraisal

All the included articles (Badawy et al. 2015; Ibrahim 2015; Karabay et al. 2016; Kaya Kara et al. 2015; Şimşek et al. 2011) were classified as level II evidence as per the NHMRC Hierarchy of Evidence (Merlin et al. 2009). The methodological quality of the five studies was assessed using a modified nine-item PEDro scale and ranged between 3/9 and 6/9, obtaining an average score of 4.4/9. Group allocation was not concealed in any of the studies, and where data were missing, these results were omitted from the analysis and no ‘intention to treat’ analysis was applied resulting in the low scores obtained.

### Description of study sample

Table 2 describes the studies’ samples included in this SR. Sample sizes across the studies ranged between 15 and 19 participants in both control and experimental groups. The mean age across studies ranged from 12.6 months to 9 years and 7 months. Three of the studies included both male and female participants (Badawy et al. 2015; Kaya Kara et al. 2015; Şimşek et al. 2011), while Ibrahim (2015) and Karabay et al. (2016) did not specify allocation by gender. Ibrahim (2015), Badawy et al. (2015) and Karabay et al. (2016) only included children with spastic diplegic CP in their respective studies.

**TABLE 2:** Description of the included studies’ sample demographics.

Variable	Type	Kaya Kara et al. (2015)	Şimşek et al. (2011)	Ibrahim (2015)	Karabay et al. (2015)	Badawy et al. (2016)
Sample size	Kinesio taping	15	15	15	15	19
	No taping	15	15	15	15	19
Gender of participants	Kinesio taping	8 males 7 females	8 males 7 females	Not specified	Not specified	10 males 9 females
	No taping	7 males 8 females	10 males 5 females			11 males 8 females
Age of participants (years)	Kinesio taping	Mean (SD): 9 year (2 year 3 month)	Mean (SD): 8 year 3 month (3 year 4 month)	Mean (SD): 8 year 4 month (1 year 9 month)	Mean (SD): 12.7 month (1.46 month)	Mean (SD): 78.05 month (28.75 month)
	No taping	Mean (SD): 9 year 7 month (3 year 4 month)	Mean (SD): 6 year 9 month (2 year 10 month)		Mean (SD): 12.6 month (1.3 month)	Mean (SD): 68.4 month (28.8 month)

*n*, number of participants; SD, standard deviation.

Badawy et al. (2015) further specified that the participants were unable to sit independently, classifying all their study participants as level IV according to the GMF Classification Scale (GMFCS). In the study by Şimşek et al. (2011), participants were rated as level III, IV or V and included children with spastic type and/or hypotonic diplegia and/or quadriplegia. Participants in Kaya Kara et al.’s (2015) study were all diagnosed with unilateral spastic CP classified as level I or II on the GMFCS.

### Description of interventions

All the studies made use of 5 cm Kinesio^®^ tape (Table 3). In the studies by Şimşek et al. (2011) and Ibrahim (2015), the KT was applied according to a fan technique along the paraspinal muscles, while Kaya Kara et al. (2015) made use of ‘I’ taping for scapular stabilisation and postural control to the upper limb or scapular area and lower limbs. Similarly, Karabay et al. (2016) made use of ‘I’ taping, extending from the acromioclavicular joint obliquely along the paraspinal area to the T12-level, while Badawy et al. (2015) applied the KT obliquely over the scapula and vertically along the paraspinal musculature. The control groups in all the studies participated in physiotherapy programmes, which included NDT and/or various upper limb, balance and functional exercises, although the exact treatment techniques differed among the studies (Table 3).

**TABLE 3:** Description of intervention (kinesiology tape and physiotherapy).

Type	Kaya Kara et al. (2015)	Şimşek et al. (2011)	Ibrahim (2015)	Badawy et al. (2016)	Karabay et al. (2015)
	Kinesio® tape (KT)	Kinesio® tape (KT)	Kinesio® tape (KT)	Kinesio® tape (KT)	Kinesio® tape (KT)
**Kinesiology taping (KT)**					
Intervention application	‘I’ taping for scapula stabilisation and postural control, using 5 cm tape (KT was also applied to lower and upper limbs)	KT was applied longitudinally between C7 and S1 along the paraspinal musculature. KT was applied from insertion to origin for children with hypertonus in trunk musculature and from origin to insertion for children with trunk hypotonia. Fan technique was applied using 5 cm KT		Two strips were placed immediately lateral to the vertebral spinous processes in a caudal-cephalo direction from the levels of L3/L4-T1. The other two strips were placed along the lower trapezius muscle from the acromion process to T12 in an oblique manner	KT tape was cut into ‘I’ strips and secured onto the acromioclavicular joint without stretch. Tape was then applied in an oblique manner to T12 with stretch, and secured at the last 5 cm without stretch
Physiotherapy management	Neurodevelopmental treatment (NDT) which consisted of stretching, weight-bearing, functional reaching and walking	Exercises focusing on tone regulation, activities of upper extremity like grabbing-releasing and activities of sitting and balance reactions related to sitting	Exercises to improve the sitting and standing position, to increase sitting and standing balance, and activities to improve the upper extremity function including reaching, grasping and release	NDT which included facilitation of rolling, sitting positions, active trunk control exercises, improving sitting balance, righting and equilibrium reactions, weight bearing exercises, hand function exercises and proprioceptive training	NDT (non-specified)
Duration	KT was applied for 12 weeks in all studies. KT was applied for 3 days after which the tape was removed for a 24-h resting period before reapplication for a further 3 days				KT was applied bilaterally for 4 weeks and was changed every 3–4 days
**No taping**					
Physiotherapy management	NDT which consisted of stretching, weight-bearing, functional reaching and walking	Exercises focusing on tone regulation, activities of upper extremity like grabbing-releasing and activities of sitting and balance reactions related to sitting	Exercises to improve the sitting and standing position, to increase sitting and standing balance, and activities to improve the upper extremity function including reaching, grasping and release	NDT which included facilitation of rolling, sitting positions, active trunk control exercises, improving sitting balance, righting and equilibrium reactions, weight bearing exercises, hand function exercises and proprioceptive training	NDT (non-specified)
Duration	Two sessions a week for 12 weeks	1-hour sessions, three times a week for 12 weeks		1.5-hour sessions, three times a week for 12 weeks	Four to five sessions per day for 4 weeks

Note: Dosage and duration of physiotherapy management in the KT group were the same as for the control group.

C1, first cervical vertebra; cm, centimetres; S1, first sacral vertebra; T12, 12th thoracic vertebra; L3, third lumbar vertebra; L4, fourth lumbar vertebra; L5, fifth lumbar vertebra.

### Description of outcome measures

Different outcome measures were utilised to assess GMF across the studies (Table 4). All the studies performed assessments at baseline and at 12 weeks, except Karabay et al. (2016), who reassessed after 4 weeks.

**TABLE 4:** Timeline and outcome measures used by included studies.

Outcome measures	Kaya Kara et al. (2015)	Şimşek et al. (2011)	Ibrahim (2015)	Badawy et al. (2016)	Karabay et al. (2015)	Testing period
Gross Motor Function Measure (GMFM)	→	→	→	→	→	Baseline
	-	-	-	-	→	4 weeks
	→	→	→	→	-	12 weeks
Sitting Assessment Scale (SAS)	-	→	-	-	-	Baseline
	-	→	-	-	-	12 weeks
Bruinisks–Oseretsky Test of Motor Proficiency (BOTMP)	→	-	-	-	-	Baseline
	→	-	-	-	-	12 weeks

The GMFM is an observational assessment tool incorporating 88 items, scored on a four-point ordinal scale (ranging from 0 to 3) (Russell et al. 2000). It is further subdivided into five domains: (A) lying and rolling, (B) sitting, (C) crawling and kneeling, (D) standing and (E) walking, running and jumping. A total score is calculated by combining the scores of all the subsections. Ibrahim (2015), Şimşek et al. (2011), Badawy et al. (2015) and Karabay et al. (2016) reported on domain (B); Kaya Kara et al. (2015) and Karabay et al. (2016) reported on domain (D); and Kaya Kara et al. (2015) reported on domain (E). Şimşek et al. (2011) was the only study that reported on total scores.

The SAS, as utilised by Şimşek et al. (2011), is a standardised observational instrument designed for the assessment of sitting in children with CP (Knox 2002). The scale is composed of five items evaluating head, trunk and foot control and arm and hand function.

The BOTMP is a standardised norm-referenced measure used to assess GMF (Bruininks & Bruininks 2010) and was only used by Kaya Kara et al. (2015). This test is used to describe motor problems of children aged between 4 years 6 months and 14 years 6 months. The BOTMP consists of eight subtests, containing 46 items, which are used to calculate a gross motor and fine motor component. The test combines gross motor skills (running speed and agility, balance, bilateral coordination and strength) and fine motor skills (upper limb coordination, response speed, visual-motor control and upper limb speed and dexterity).

### The effect of kinesiology taping versus no taping on gross motor function

The effect of KT as an adjunct to physiotherapy on GMF in children with CP is summarised in the tables and/or forest plots.

#### Gross motor function: Sitting (Gross Motor Function Measure [B])

Ibrahim (2015), Şimşek et al. (2011) and Badawy et al. (2015) found a significant difference between baseline and 12 weeks for both the KT and non-taping groups. When comparing the changes between the two groups at the end of the 12-week period, both Ibrahim (2015) and Badawy et al. (2015) found a significant difference favouring the KT group with *p* = 0.005 and *p* < 0.05, respectively (Table 5). Comparable results were found in the study conducted by Karabay et al. (2016), favouring the KT group. The only difference was that this study was conducted over a 4-week period compared to 12 weeks in Ibrahim (2015), Şimşek et al. (2011) and Badawy et al. (2015). Both the KT and non-taping group improved significantly between baseline and 4 weeks. When the results were compared at the end of the 4-week period, a significant difference was found in favour of the KT group (*p* < 0.01).

**TABLE 5:** Effect of kinesiology taping on gross motor function as determined by the Gross Motor Function Measure (B).

Reference	Outcome measure	No taping	KT	*p*-value for between-group effect
Ibrahim (2015)	GMFM (B) – baseline	34.84 (8.40)	35.85 (7.25)	0.005*
	GMFM (B) – 12 weeks	42.48 (9.21)	49.90 (2.11)	
	*p*-value (within-group effect)	0.020*	< 0.001*	-
Şimşek et al. (2011)	GMFM (B) – baseline	57.97 (24.60)	57.10 (24.30)	0.127
	GMFM (B) – 12 weeks	61.66 (22.56)	75.66 (25.12)	
	*p*-value (within-group effect)	0.011*	0.001*	
Badawy et al. (2016)	GMFM (B) – baseline	29.85 (3.5)	29.76 (3.4)	< 0.050*
	GMFM (B) – 12 weeks	45.37 (3.2)	69.86 (4.1)	
	*p*-value (within-group effect)	< 0.050*	< 0.050*	-
Karabay et al. (2015)	GMFM (B) – baseline	39.30 (14.4)	34.20 (16.5)	< 0.010*
	GMFM (B) – 4 weeks	43.70 (14.5)	41.00 (15.5)	
	*p*-value (within-group effect)	0.000*	0.000*	-

GMFM (B), the (B) describes the sitting domain evaluated in GMFM; GMFM, Gross Motor Function Measure; KT, kinesiology taping.

* , Values that indicate statistically significant results.

**FIGURE 1:**
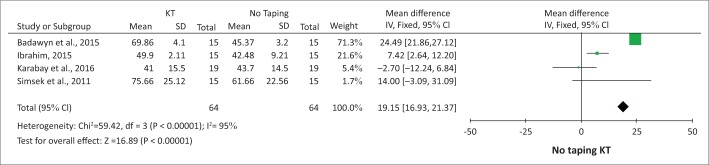
Kinesiology taping versus no taping as measured by Gross Motor Function Measure (B) sitting function at the end of the intervention period.

A meta-analysis illustrates KT to be favoured for GMFM (B) (*p* < 0.0001) when compared to no taping in improving sitting function in children with CP (Figure 1).

Şimşek et al. (2011) utilised the GMFM (B) and SAS to determine the effect of KT on sitting function. Both outcome measures showed a significant difference between baseline and 12 weeks for the KT and no taping groups. However, when the results were compared at the end of 12 weeks, only the SAS showed a significant improvement (Table 6).

**TABLE 6:** Effect of kinesiology taping on sitting function as determined by Gross Motor Function Measure (B) and Sitting Assessment Scale.

Reference	Outcome measure	No taping mean (SD)	Kinesiology taping mean (SD)	*p*-value for between-group effect
Şimşek et al. (2011)	GMFM (B) – baseline	57.97 (24.60)	57.10 (24.30)	0.925
	GMFM (B) – 12 weeks	61.66 (22.56)	75.66 (25.12)	0.127
	*p*-value (within-group effect)	0.011*	0.001*	-
Şimşek et al. (2011)	SAS – baseline	12.47 (3.64)	13.53 (3.48)	0.419
	SAS – 12 weeks	13.20 (3.32)	16.47 (1.96)	0.003*
	*p*-value (within-group effect)	0.028*	0.000*	-

GMFM (B), the (B) describes the sitting domain evaluated in GMFM; GMFM, Gross Motor Function Measure; SAS, Sitting Assessment Scale; SD, standard deviation.

* , Values that indicate statistically significant results.

#### Gross motor function: Standing (Gross Motor Function Measure [D])

Both Ibrahim (2015) and Kaya Kara et al. (2015) showed a significant improvement from baseline to 12 weeks for the KT group. However, when comparing the KT to the no taping group at the end of 12 weeks, only Ibrahim (2015) shows a significant difference favouring the KT group (*p* = 0.003) (Table 7).

**TABLE 7:** Effect of kinesiology taping on standing function as determined by Gross Motor Function Measure (D).

Reference	Outcome measure	No taping mean (SD)	KT mean (SD)	*p*-value for between-group effect
Ibrahim (2015)	GMFM (D) – baseline	28.73 (5.76)	27.11 (1.45)	-
	GMFM (D) – 12 weeks	33.23 (4.83)	37.85 (2.82)	0.003*
	*p*-value (within-group effect)	0.020*	0.0001*	-
Kaya Kara et al. (2015)	GMFM (D) – baseline	-	-	-
	GMFM (D) – 12 weeks	1.37 (3.47)	3.23 (4.88)	0.239
	*p*-value (within-group effect)	0.684	0.028*	-

GMFM (D), the (D) describes the standing domain evaluated in GMFM.

GMFM, Gross Motor Function Measure; KT, kinesiology taping; SD, standard deviation.

* , Values that indicate statistically significant results.

#### Gross motor function: Bruininks–Oseretsky Test of Motor Proficiency

In Kaya Kara et al.’s (2015) study, only the KT group showed a significant improvement between baseline and 12 weeks (*p* = 0.025). When the results were compared at the end of the intervention period, they showed a significant change favouring the KT group (*p* = 0.019) (Table 8).

**TABLE 8:** Effect of kinesiology taping on standing as determined by Gross Motor Function Measure (E).

Reference	Outcome measure	No taping mean (SD)	KT mean (SD)	*p*-value for between-group effect
Kaya Kara et al. (2015)	GMFM (E) – baseline	-	-	0.818
	GMFM (E) – 12 weeks	0.94 (1.81)	2 (2.12)	0.227
	*p*-value (within-group effect)	0.036*	0.005*	-

GMFM (E), the (E) describes the walking, running and jumping domain evaluated in GMFM; GMFM, Gross Motor Function Measure; KT, kinesiology taping; SD, standard deviation.

* , Values that indicate statistically significant results.

## Discussion

Kinesiology taping is a treatment technique increasingly being used in numerous fields of physiotherapy to improve posture and function (Jaraczewska & Long 2006; Şimşek et al. 2011; Yasukawa, Patel & Sisung 2006) although it is reported only to be effective for as long as the KT remains on the skin (Thelen et al. 2008). This is the first SR to report on the effectiveness of KT applied to the trunk in children with CP. The data were comparable because of all studies utilising the same outcome measure, namely the GMFM. Furthermore, four of the five studies tested the participants at similar time intervals and all studies used the same kinesiology-type taping. Studies did differ in terms of how the tape was applied to the trunk. Contrary to the inconclusive findings from Cunha et al.’s (2017) and Guchan and Mutlu’s (2016) SRs, this study was able to show that truncal application of KT is an effective adjunct to physiotherapy to improve GMF in children with CP. Children with spastic diplegia and higher functioning children, classified as level I or II according to the GMFCS, seem to benefit more than lower functioning children.

Sitting function as determined by GMFM (B) in three out of the four studies reported a significant overall effect in favour of the KT group (Badawy et al. 2015; Ibrahim 2015; Karabay et al. 2016). The one study (Şimşek et al. 2011) that reported no effect, despite applying the KT using the same method as used by Ibrahim (2015) (along the paravertebral musculature for 12 weeks), included children with spastic quadriplegia, who functioned mainly at GMFCS level III or V, while the other three studies only included children with spastic diplegia. This was supported by the fifth study included in this review (Kaya Kara et al. 2015), which found that GMFM scores tended to reach their highest levels in CP children with higher motor abilities.

Despite the lack of significant change on the GMFM (B), Şimşek et al. (2011) did show significant improvement in the KT group when using the SAS. This could be explained in that the GMFM (B) measures the ability to maintain a sitting position while changing the centre of gravity in and out of the base of support, whereas the SAS measures head, trunk and foot control as well as arm and hand function. The SAS seems to be more sensitive for detecting changes in sitting balance because it measures a wider range of functional activities in the seated position compared to the GMFM (B).

Measurement of the kyphotic angle was an additional outcome measured by Badawy et al. (2015) and Karabay et al. (2016) to determine the effect of KT on posture. Both these studies reported a significant reduction in the kyphotic angle. Interestingly, a larger improvement was reported by Badawy et al. (2015) and could be attributed to the increased intervention period of 12 weeks when compared to the 4 weeks in Karabay et al. (2016). It can be hypothesised that KT should be applied for a longer period of time, which, in turn, will reduce the kyphotic angle and improve postural alignment, therefore increasing the potential for improving sitting function.

Although pooling of data via meta-analysis favoured the KT group, the effect on standing posture and function was, however, contradictory. One study found that KT improved standing function (Karabay et al. 2016), whereas another reported no significant improvement as determined by the GMFM (D) (Kaya Kara et al. 2015). Both studies included children with similar classifications (GMFSC levels I and II). The difference in outcome between these two studies could be explained by the method of taping – one study used an ‘I’ taping technique for scapular stabilisation and postural control (Kaya Kara et al. 2015), whereas the other study applied KT longitudinally along the paraspinal musculature between C7 and S1 (Karabay et al. 2016). Although both applications are aimed at improving thoracic extension affecting postural control, the ‘I’ technique also incorporates the scapular stabilisers. Despite this, however, it seems the paraspinal taping method that extends further down the spine is more effective. This would, however, need further investigation.

One study included additional measures for GMF and also reported on BOTMP and GMFM (E) scores (Kaya Kara et al. 2015). These measures, performed within the same sample, demonstrated different outcomes for walking, running and jumping. One would expect similar results to be found for the same functional activity. The BOTMP is more commonly used to measure motor performance in children with typical development or presenting with minimal motor dysfunction such as developmental coordination disorder (DCD) (Bruininks & Bruininks 2010), whereas the GMFM was developed specifically for determining functional ability in children with CP (Russell et al. 2000). Although deemed valid for use in a CP population, the BOTMP includes more functional parameters (Kaya Kara et al. 2015), which may have allowed for detection of smaller effect sizes in walking, running and jumping. Given that no change was recorded using the GMFM from pre- to post-KT application in their study, it may be that the small effect sizes are not clinically significant in children with CP.

### Limitations

This review limited its investigation of taping to KT. Other forms, such as rigid and therapeutic taping as described by Footer (2006) and Iosa et al. (2010), were not included because of the difference in taping properties and potential resultant effect. A methodological limitation was that the RCTs included in this review were restricted to English, which may have resulted in otherwise eligible foreign publications not being included. Unpublished data were also not included which may have led to publication bias, especially if these included studies that resulted in an unfavourable outcome.

Despite only RCTs being included in this review, the methodological quality of these studies was low to moderate, with PEDro scores ranging from 3 to 6 out of 9. Scores were lost regarding the issue of blinding. The nature of the intervention, however, does not allow for blinding of neither the participant nor the therapists administering the intervention. However, therapists may have altered their physiotherapy intervention knowing which children were receiving the investigational intervention. Sham taping could possibly have reduced treatment bias. In addition, the assessor was blinded in only one study (Kaya Kara et al. 2015) as to which arm of the study participants were allocated. This may have introduced assessor bias into the other studies with an impact on the review’s study outcomes. Similarly, although all included studies reported randomised allocation, this was not concealed. Another criterion that scored low on the PEDro scale was that none applied ‘intention to treat’ analysis in cases where the data were missing. These all may have led to exaggerated estimates of the treatment effects.

One of the main strengths of this SR was that the full spectrum of children as classified by the GMFCS was included. Although our findings suggest that there is moderate evidence that taping is more effective in higher functioning children, this is based on the outcome of only four studies, and in one (Karabay et al. 2016), it is not clear how comparable the intervention and control groups were at baseline. Similarly all the studies included in this review had relatively small sample sizes. Although sample size calculations were performed in some studies and their sample sizes considered appropriate to determine effect, it did not allow for subgroup analysis. Larger sample sizes would allow more clarity on which classification and diagnosis of CP would benefit more from KT.

All studies only reported on the effect of KT after 4 or 12 weeks of intervention. There was no description of immediate or long-term effects of KT in this population. The current evidence suggests that the lasting effect beyond physiotherapy intervention is limited to as long as the KT is applied (Thelen et al. 2008). Without description of the immediate effect for the application of the tape, it is difficult to interpret or conclude on the appropriateness of a 12-week duration of the intervention. Similarly, there is no evidence to suggest that following removal of the tape, the changes reported in function can or will be maintained.

### Clinical relevance and recommendations

It is recommended that the type of application of KT be considered when using this as an adjunct to physiotherapy treatment, as described by the studies. Although KT is relatively inexpensive, the tape needs to be replaced every 3 days which, in a poorly resourced setting, can become quite costly. However, it can be applied by any trained therapist and is usually readily accessible, which makes KT a favourable intervention to use.

It is also recommended that further studies investigate the long-term effects of KT, both regarding how well and how long application can be tolerated by the skin and whether the functional gains achieved during the exposure period are maintained in the long term. Blinding assessors (and where possible blinding the therapist) is also recommended to avoid treatment and measurement bias. We also propose *n* = 1 studies to assist in better understanding of KT to address individual needs of children with CP given their varied presentations and functional abilities. This, in turn, will facilitate decision-making as to its utility in differing clinical settings and contexts.

## Conclusion

Kinesiology taping applied to children with spastic diplegia and/or functioning at GMFCS levels I and II has moderate evidence (level II) for effectiveness in improving GMF when used as an adjunct to physiotherapy. Clinicians will find it most beneficial on children who require improved sitting postural control to perform functional tasks. Further research is required to better understand the short- and long-term effects of KT. It is also recommended that the type of application of KT be considered when using this as an adjunct to physiotherapy treatment.
